# The League of Remembrance

**Published:** 1923-08

**Authors:** 


### THE LEAGUE OF REMEMBRANCE.

### ITS WORK FOR HOSPITALS.

The League of Remembrance was founded to carry on certain war services for the hospitals which were formerly discharged by Queen Mary's Needlework Guild. Mr. John Murray, the Chairman of the League, presiding at its commemmoration dinner on July 11th at the Connaught Rooms, said that the suggestion of consolidating all the admirable work called up by the war, and the bringing together of those who were interested to provide the nucleus of a great beneficent body, came from the late Duchess of Albany. The League of Remembrance is the outcome of that idea.

It has a particular claim upon all those interested in hospitals, since its first object is to continue its war work in times of peace by supplying medical and surgical requisites and clothing to naval, military and civil hospitals and other institutions and organisations engaged in the promotion of health or social welfare. The materials are provided by the organisations requiring assistance, and the League makes them up into whatever dressings or garments that may be required, making no charge for its services.

Viscount Burnham, who is one of the patrons of the League, made an interesting suggestion in a speech at the dinner on "National Service." The League, he said, embodied the best ideals of service and sacrifice. To meet the normal needs of our hospitals there was a greater necessity than ever for beneficent activities in the supply of the medical and surgical requirements for which the League existed. It would be a happy thing, he thought, if the whole of our hospitals and centres of infant welfare were supplied with articles equal in quality to those which came from the League. Nor could Lord Burnham see why the organisation should not be coextensive with the whole of our hospital system, since it was rooted in the same great tradition of voluntary service and voluntary management.

Princess Beatrice, the President of the League, and Princess Alice, Countess of Athlone, were present at the dinner. Other speakers were Mr. Horace Annesley Vachell, Major Ian Hay Beith ("Ian Hay") and Mr. W. R. Dunlop of the Colonial Service in the British West Indies. Mr. Dunlop said that the West Indies had a small organisation in London connected with infant welfare, but it was about to be wound up for lack of support and patronage. He thought that the League of Remembrance had great possibilities of doing Empire work by affiliating such small bodies, and so preventing their labours from being brought to a standstill.

### Sanitary Association of Scotland.

The "Transactions of the Incorporated Sanitary Association of Scotland" include the Annual Report of the Council a register of members, and a detailed account of the Association's Annual Congress last year at Rothesay. Two examinations in sanitary science have been conducted by the Association, and two examinations in the inspection of meat and other foods. During the year 24 new members were admitted, but the names of 47 persons were on various grounds removed from the roll.

